# Intercalary Elbow Resection and Arthrodesis for Giant Cell Tumor of Distal Humerus: Something Is Better Than Nothing

**DOI:** 10.7759/cureus.28698

**Published:** 2022-09-02

**Authors:** Umesh Yadav, Mudit Nemani, Manmeet Malik, Shagnik Paul, Amandeep Mittal, Gaurav K Agrawal, Nishan Yadav

**Affiliations:** 1 Orthopedics, Pandit Bhagwat Dayal Sharma Post Graduate Institute of Medical Sciences, Rohtak, IND; 2 Orthopedics and Trauma, Pandit Bhagwat Dayal Sharma Post Graduate Institute of Medical Sciences, Rohtak, IND

**Keywords:** gct, gct treatment, wide resection, bone tumour and limb salvage, elbow arthrodesis, giant-cell tumor of bone

## Abstract

Giant cell tumor (GCT) of the bone is a benign, locally aggressive neoplasm of epiphyseal origin. Most common sites for GCTs include the distal femur, proximal tibia, and the distal end of radius with the distal humerus being involved rarely. GCT is predominantly managed by extended curettage followed by adjuvant therapy to reduce recurrence. Juxta-articular GCTs are difficult to manage due to the destruction of the articular cartilage and subchondral bone which necessitates the need for joint reconstruction or fusion to salvage the joint. Aggressive and recurrent GCTs can be managed by wide resection of the tumor to reduce local recurrence followed by joint reconstruction or fusion. Joint reconstruction using a total elbow arthroplasty has been described for limb salvage as it provides a good functional outcome. We present a case of an aggressive GCT of the distal humerus that was treated using wide resection with humero-ulnar arthrodesis as an alternative in situations where joint reconstruction is not possible due to the unavailability of the prosthesis or socio-economic factors. The patient was asymptomatic after two years of follow-up, had no signs of recurrence, and had good hand functions.

## Introduction

Giant cell tumor (GCT) is a usually benign, locally aggressive primary bone tumor. It is metaphyseal-epiphyseal in origin and is predominantly found in the distal end of the femur, distal end of the radius, and the proximal end of the tibia. It may also be seen in the pelvis, spine, and the small bones of hands and feet. Elbow remains a rare site for GCT. It appears as an eccentrically located, expansile, lytic lesion in skeletally mature individuals most commonly between the third and fourth decades of life [1].

The treatment most commonly employed for GCTs is an extended intra-lesional curettage followed by the use of bone cement and adjuncts such as phenol or alcohol. The ideal treatment for this tumor has not been achieved despite extensive studies [2]. Studies report more than 50% local recurrence after simple curettage. Extended curettage is most commonly done for GCTs using chemical cauterization with alcohol and phenol, liquid nitrogen, use of high-speed burrs, pulsatile lavage, and thermocoagulation which is followed by filling of the defect with bone grafts, bone graft substitutes, or polymethylmethacrylate (PMMA) [2-5]. This procedure is reported to have a recurrence of 0%-25% [3-6]. En-bloc resection has been advocated to avoid local recurrence [4-6]. However, such cases may require arthrodesis or joint replacement due to their juxta-articular nature. Campanacci grade 3 tumors with extension into the joint or surrounding soft tissues and cases with less than two-thirds intact circumferential cortical bone require wide excision and joint reconstruction. This reduces the local recurrence to around 5% [6,7]. We present a case of aggressive GCT of the distal humerus treated by en-bloc resection and humero-ulnar arthrodesis.

## Case presentation

**Figure 1:**
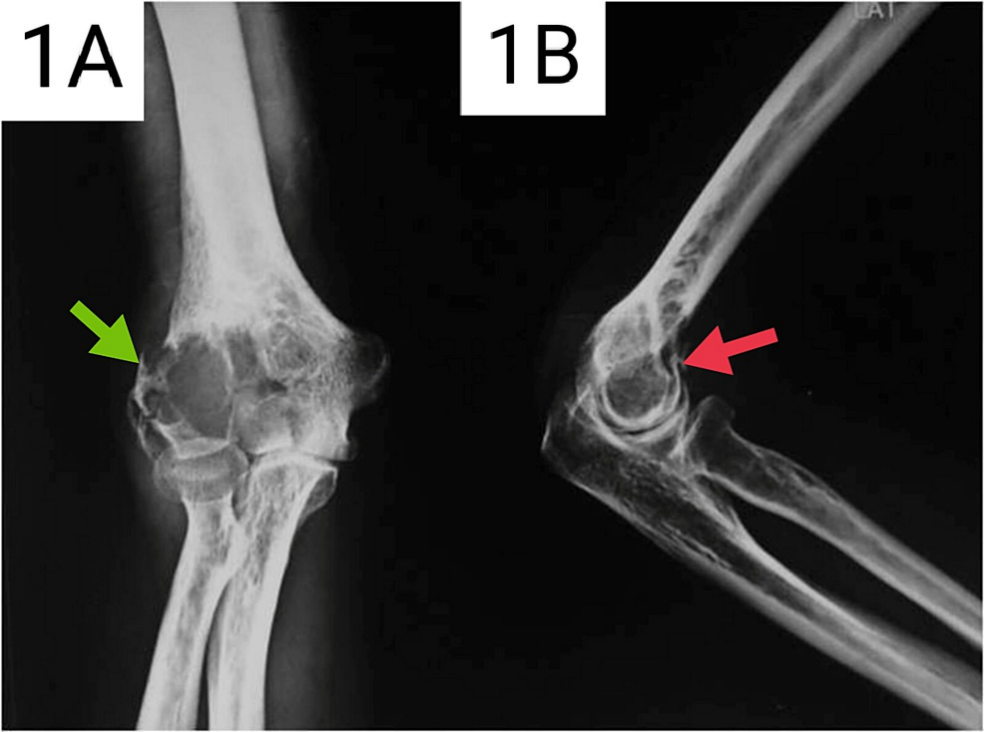
(A) Anteroposterior radiograph of the right elbow at the time of initial presentation. (B) Lateral radiograph of the right elbow at the time of presentation. Green arrow - Lytic lesion with "soap bubble" appearance. Red arrow - Lytic lesion in the distal humerus

A 30-year-old male presented to the Outpatient Department (OPD) with a history of pain, swelling, and decreased range of motion of the right elbow for six months. There was no preceding history of trauma. On examination, there was an increase in the local temperature and tenderness around the right elbow. There was a flexion deformity of 30º with a painful range of motion of 30º to 90º. Pronation and supination were restricted due to pain. Radiographs showed an expansile lytic lesion in the distal humerus with a classical “soap-bubble” appearance. A magnetic resonance image (MRI) was performed which showed a well-defined lesion of the distal humerus with altered signal intensity on both T1 and T2 weighted images, breaching the lateral cortex of the distal humerus. A biopsy was taken which confirmed the diagnosis to be a GCT of the distal humerus. Surgery was advised; however, the patient did not opt for the procedure. Figures 1A, 1B show the anteroposterior and lateral radiographs of the patient at the time of presentation.

The patient visited the OPD again after one year with increased pain, swelling, and gross restriction of movements of the right elbow. The elbow was in 50º of flexion with further flexion up to 80º. There was a swelling of 4x5cm over the lateral portion of the elbow, which was tender, nonmobile, and firm in consistency, with an irregular surface and irregular margins. The skin over the swelling was inflamed and engorged veins were seen. The patient complained of numbness over the lateral part of the dorsum of the hand, and wrist drop indicating an involvement of the radial nerve. Radiographs showed an expansile osteolytic lesion involving the lateral condyle, part of the trochlea, and medial condyle extending proximally with a breach of the lateral cortex and soft tissue shadows. MRI showed a breach of the lateral cortex with the tumor extending into the soft tissue. The radial nerve was found to be entrapped in the tumor mass. The elbow joint was found to be involved. Computed tomography (CT) did not show evidence of pulmonary metastasis. The tumor was graded as Campanacci Grade III GCT.

**Figure 2:**
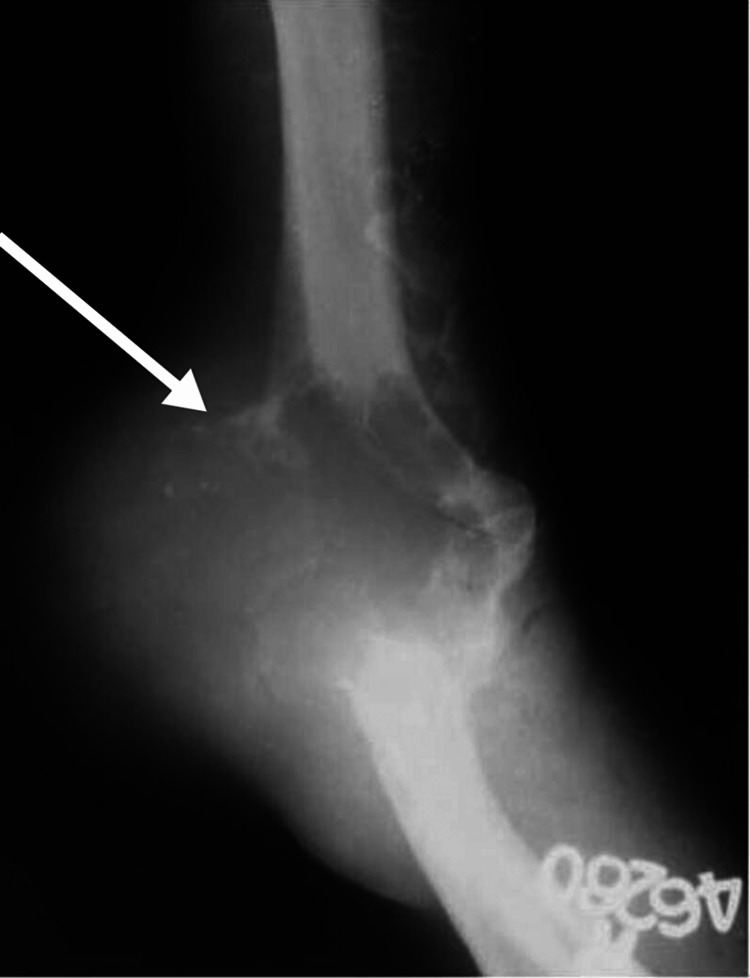
Anteroposterior radiograph of the right elbow after one year. White arrow - Expansile, lytic lesion with cortical breach and soft tissue involvement

**Figure 3:**
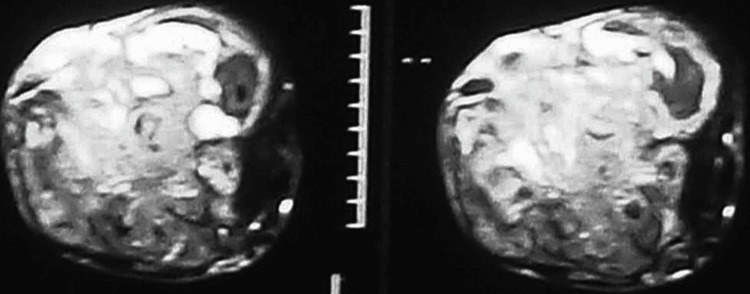
MRI image of the right elbow showing entrapment of the radial nerve in the substance of the tumor

Figure 2 shows the anteroposterior radiograph of the patient when he presented after one year. Figure 3 shows the MRI of the patient with radial nerve entrapment in the tumor substance.

 

After explaining the risks and taking an informed consent, the patient was taken for surgery. The patient underwent intercalary resection of the lower arm with tumor and upper forearm. The tumor was completely excised and humero-ulnar arthrodesis was done. Due to the lack of availability of suitable implants, humero-ulnar arthrodesis was done in extension. This provided greater stability. The radial nerve was resected with tumor allowing for adequate margins. The remaining neurovascular structures were identified and preserved. The limb was immobilized in extension with an above elbow plaster back slab. The postoperative period was unremarkable. Tendon transfers were performed one year after surgery for the wrist drop after ensuring that there was no recurrence. Jones tendon transfers was performed. After three months of physiotherapy the patient had good hand functions and was satisfied with the functional results. After two years of follow-up, the patient was asymptomatic with no recurrence and good hand functions.

**Figure 4:**
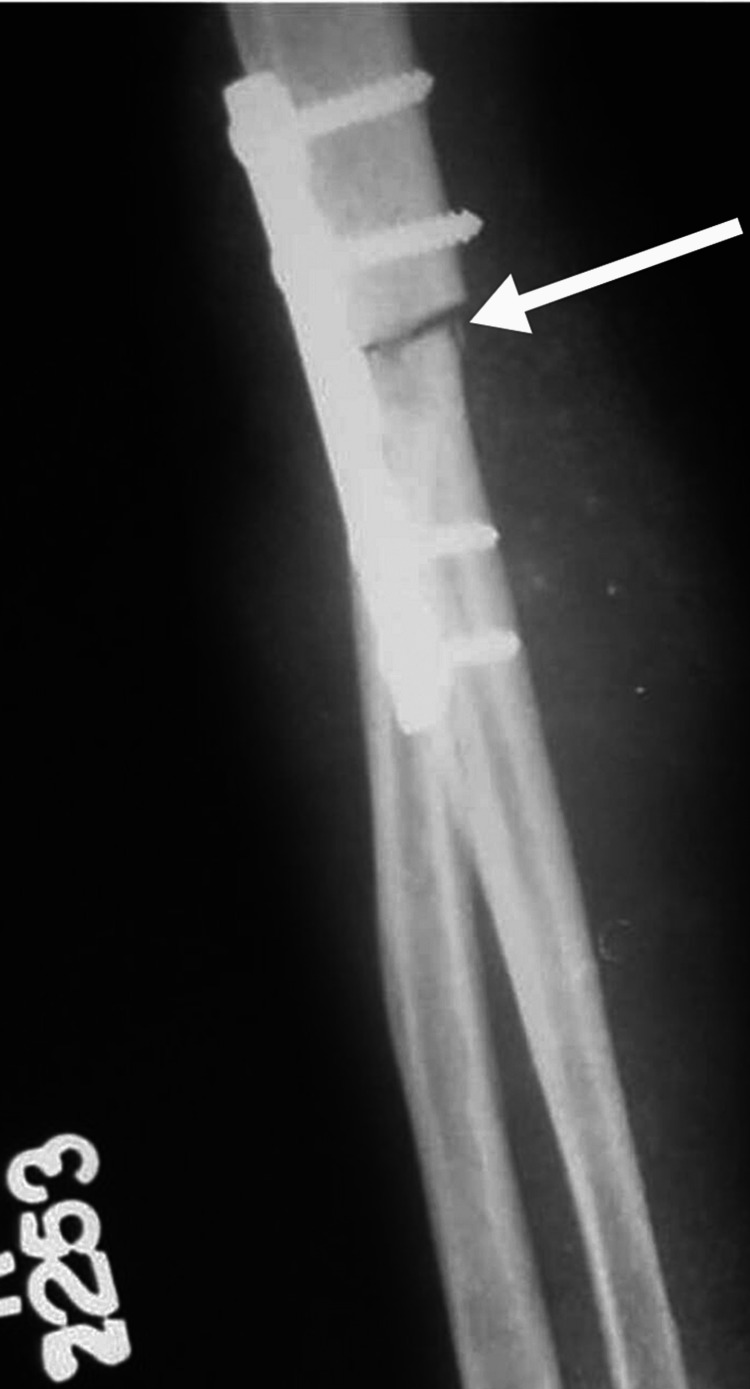
Postoperative radiograph showing humero-ulnar arthrodesis White arrow - hmero-ulnar arthrodesis using a plate

**Figure 5:**
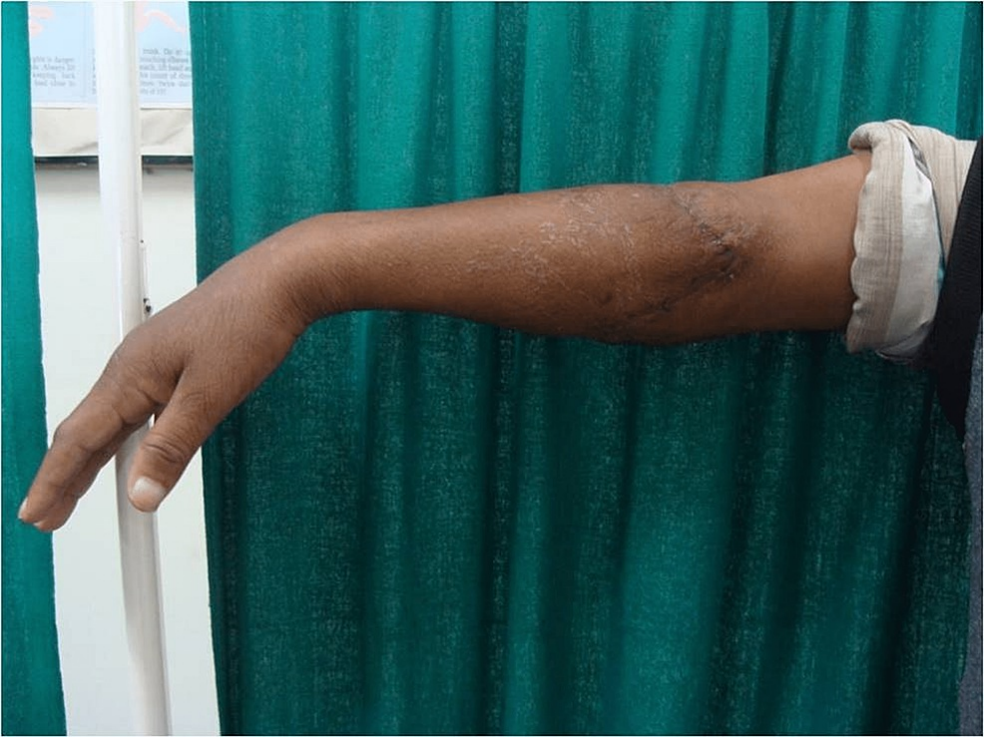
Right elbow of the patient after two years of follow up.

**Figure 6:**
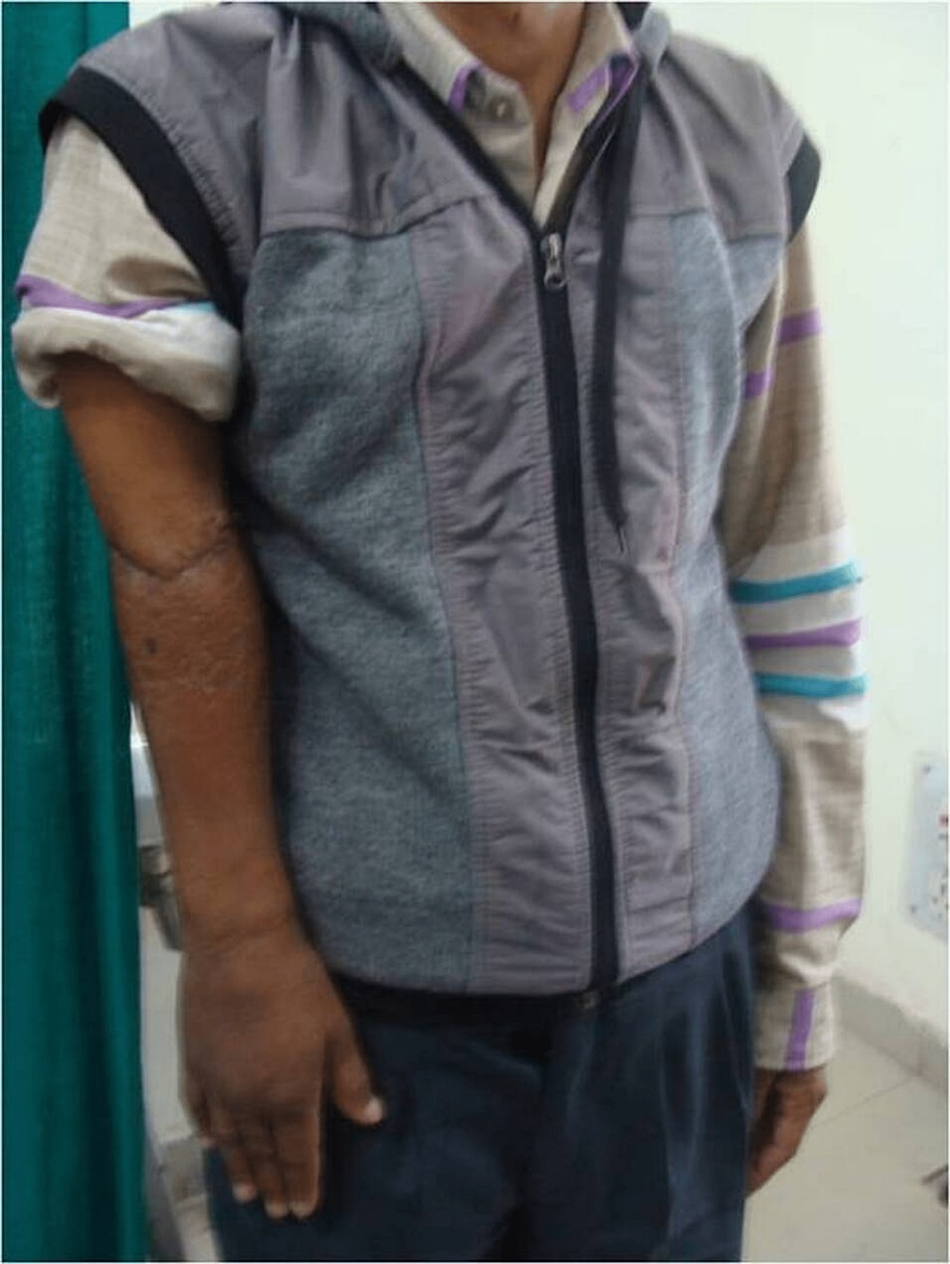
Right elbow of the patient after two years of follow-up showing minimal shortening as compared opposite limb.

Figure 4 shows an anteroposterior view of the right elbow after wide resection and humero-ulnar arthrodesis. Figures 5, 6 show the clinical picture of the patient after two years of surgery.

## Discussion

GCTs are mostly treated by extended curettage followed by bone grafting, cementing, or both. However, more aggressive forms of GCT treated by intralesional curettage have a higher risk of recurrence. Even with the inferior functional outcome as compared to intralesional curettage, wide resection is preferred in aggressive tumors and patients with recurrence. Wide local resection with total elbow arthroplasty restores the range of motion of the elbow, lowers the risk of recurrence, and lower complication rates. Literature is filled on the treatment of GCT with total elbow arthroplasty [8-10].

Hemiarticular and total elbow allografts can be used as salvage procedures after failed total elbow arthroplasty. Financial concerns may limit the easy availability of implants in a developing country like India. Although reconstruction with prostheses and allografts provides a good functional outcome in the short term, they may not be considered ideal for young active patients with a normal life expectancy. Such patients may require revision surgeries as they are likely to outlive the expected life of such a hinged prosthesis. Moreover, radiotherapy and chemotherapy used for control of certain tumors, are associated with unpredictable outcomes regarding the union. Autografts used, are not subject to such unpredictability. Thus, arthrodesis provides a viable option in such cases [11].

Kapoor et al. used surgical phocomelia as a limb salvage procedure for patients with extensive sarcoma of the upper limb and found it to be an effective method of treatment and better than amputation in terms of the outcome [12]. Hahn et al. analyzed segmental resection and replantation in six patients with malignant bone tumors including two patients with GCTs and found it to be an effective partial limb salvage method. It was postulated that in certain circumstances, segmental resection of the tumor-bearing region in a cylindrical fashion and replantation of the distal part of the limb with a microsurgical technique might be a suitable alternative that would provide better functional outcomes as compared to amputation. This procedure would work along the same principles as rotationplasty [13].

The indication for resection-replantation as partial limb salvage surgery is between that for resection reconstruction and amputation. Similar to the rotationplasty of the leg, there is considerable shortening. However, when dressed the difference in arm length is not eye-catching. Moreover, the primary concern is the oncological safety of the patient. The retention of as much function as possible is a secondary concern. It is difficult to compare the functional results of the residual limb with cases where a prosthesis was implanted or amputation due to the shortening of the limb. There may be some diminution in hand function after such a procedure. However, such results may be due to partial resection of the distal muscles and re-suturing under variable tensions depending on their residual length and need to cover the bone as well as neurovascular bundles. In situations where the nerves may be saved, good hand function may be obtained by looping and transposition of the nerves. Even a partial limb salvage is valuable for body image and function especially in aggressive cases when presented as an option to avoid amputation [14].

When compared with above-the-knee amputation or disarticulation of the hip, the use of a limb-salvage procedure such as arthrodesis or rotationplasty for osteosarcoma of the distal end of the femur did not compromise long-term survival or shorten the disease-free interval in patients. The only obstacle faced was disturbed body image [15]. This was a minor inconvenience since the function of the shortened limb is satisfactory in most cases [16].

## Conclusions

Limb salvage surgery by wide resection with humero-ulnar arthrodesis is a viable treatment option in young, active patients in aggressive GCTs. Total elbow arthroplasty is preferred as it has a better functional outcome, but elbow arthrodesis may provide a valuable alternative in poor societies and lack of availability. A major inconvenience is diminished hand function, which is, in most cases, satisfactory in cases without nerve involvement and only poses a minor inconvenience. Arthrodesis provides a better cosmetic outcome as compared to amputation of the upper limb, which is important for body image. This procedure has a higher acceptance in cases where amputation serves as the only alternative due to the lack of availability of joint reconstruction measures and in low-income societies.
